# 

**Published:** 1889-11-23

**Authors:** 


					k'lfis Jr* Ul
M Vol.. vn.-
?November 23rd. 1889,
[w ' ? ?ii.?im u v em u?r zoru, io{
tran^Sat- General Post Office as a Newspaper for
p amission in the United Kingdom and Abroad.
WITH EXTRA NURSING SUPPLEMENT.
Per Annum, 6b. 6d., post free.
Monthly Parts, 6d.; post free 7?d.
Ditto per Annum, 7s. 6d.. Dost free.

				

